# First Prospective Multicenter Evaluation of Robotic-assisted Partial Nephrectomy Using the DEXTER Robotic Surgery System

**DOI:** 10.1016/j.euros.2026.02.001

**Published:** 2026-02-18

**Authors:** Damien Thillou, Xavier Durand, Humphrey Robin, Aurélien Forgues, Nadia Ali Benali, Damien Emeriau, Guillaume Hugues

**Affiliations:** a Department of Urology, Saintes Hospital, Saintes, France; b Department of Urology, Hospital Paris Saint-Joseph, Paris, France

**Keywords:** DEXTER Robotic Surgery System, Minimally invasive surgery, Partial nephrectomy, Robotic-assisted surgery

## Abstract

**Background and objective:**

Robot-assisted partial nephrectomy (RAPN) is a standard treatment for small renal tumors due to its precision and nephron-sparing properties. The DEXTER Robotic Surgery System is an open, sterile, compact, three-arm multiport system. This study evaluated the intraoperative and early postoperative safety and clinical performance of DEXTER in RAPN.

**Methods:**

A prospective observational study was conducted at two hospitals in France. Seven surgeons performed surgeries using DEXTER. The primary endpoints were major complications (Clavien-Dindo grades III–V) and procedural success rate. The secondary endpoints included perioperative outcomes and 30-d follow-up assessment.

Key findings and limitations

Thirty-three patients with a median age of 70 yr (interquartile range [IQR] 60–74) and a body mass index of 26.2 kg/m^2^ (IQR 23.7–31.2) were enrolled. The clampless technique was used in six cases. When full (22 cases) or selective (five cases) clamping was applied, the median warm ischemia time was 20 min (IQR 14–25). All procedures were completed without conversion to open or laparoscopic surgery. Three Clavien-Dindo grade III events occurred. The median estimated blood loss was 300 ml (IQR 100–600), with no blood transfusions. The skin-to-skin operative time was 170 min (IQR 137–200), and the length of hospital stay was 2 d (IQR 2–3). Limitations included the lack of a learning curve assessment due to a low case load per surgeon, and reduced comparability due to a short follow-up time and lack of a control group.

**Conclusions and clinical implications:**

RAPN with DEXTER is feasible, and the study supports its short-term safety, even during the initial cases. Further studies are needed to assess its long-term performance.

**Patient summary:**

This is a prospective, multicenter cohort study of patients who underwent robot-assisted partial nephrectomy with the DEXTER Robotic Surgery System. During the study, all procedures were completed successfully without any conversions to open or laparoscopic surgery. The results support the short-term safety and feasibility, and highlight the ease of implementation of DEXTER. Further studies are needed to assess its long-term performance.

## Take Home Message

This first multicenter experience with DEXTER-assisted partial nephrectomy demonstrates safety, feasibility, and intuitive usability. All procedures were completed without conversions, complication rates were comparable with those of the established platforms, warm ischemia times remained safe, and the LAP mode enabled flexible robotic-laparoscopic transitions.

## 1 Introduction

Renal cell carcinoma accounts for 90% of kidney malignancies [1] with a global incidence of 4.6/100 000 in 2020 [1], [2] and mortality rate of 1.8/100 000 [3]. Partial nephrectomy is the standard treatment for small renal tumors due to its high precision and nephron-sparing properties [4]. Since the introduction of laparoscopic partial nephrectomy in 1993 and robot-assisted partial nephrectomy (RAPN) in 2004, minimally invasive techniques for partial nephrectomy have become the preferred surgical approach, thanks to the favorable oncological outcomes and low complication rates [5], [6].

Despite the advantages of robotic surgery, associated limitations hinder its adoption across health care settings. The acquisition cost is high [7], [8], and most systems are associated with a large physical space requirement [9]. Additionally, the use of already existing operating room (OR) tools is limited since laparoscopic tools are incompatible with robotic arms. Recent data indicate that, in France, approximately 20% of partial nephrectomies are still performed laparoscopically [10].

The DEXTER Robotic Surgery System is a versatile robotic platform consisting of an open, sterile surgeon console; two patient carts; and a robotic endoscope arm. The compact footprint of DEXTER facilitates transportation between rooms and seamless integration in any OR. DEXTER received a CE mark in 2020 and U.S. Food and Drug Administration approval in 2024.

The safety and feasibility of DEXTER have been demonstrated in urology, including the first robot-assisted radical prostatectomy (RARP) in 2023 [11], a case series with ten consecutive patients who underwent RARP with lymph node dissection [12], where all patients were treated successfully without any device-related complications, highlighting safety. A retrospective analysis of 47 RARP procedures confirmed the safety and feasibility of DEXTER, and additionally reported on the functional and early oncological outcomes [13]. The study further demonstrated that surgeons with no previous robotic experience could rapidly adapt to the system. DEXTER has additionally been used in gynecology [14], [15] and general surgery [16], [17], [18], [19].

The objective of this postmarket clinical follow-up (PMCF) study was to document the perioperative and early postoperative safety and usability performance of DEXTER in its first RAPN case series.

## 2 Patients and methods

This prospective, single-arm, multicenter study was conducted according to the Declaration of Helsinki, ISO 14155:2020, and applicable data protection regulations. The study, registered in the ClinicalTrials.gov database (NCT05537727), was part of PMCF data collection for the CE-marked DEXTER Robotic Surgery System (Distalmotion SA, Switzerland), used within its intended purpose and clinical indications. The PMCF full study included hysterectomy, partial nephrectomy, and right colectomy. This article reports data from the partial nephrectomy cohort recruited at two hospitals in France. The study was noninterventional and did not require any additional interventions or burdens beyond standard clinical care.

Eligible patients were aged ≥18 yr, able to provide informed consent, agreed to 30-d follow-up, and were scheduled to undergo RAPN for renal tumors. The exclusion criteria included body mass index (BMI) ≥40 kg/m^2^, contraindications to endoscopic surgery, bleeding diathesis, pacemakers or internal defibrillators, pregnancy, concomitant procedures, and participation in another clinical trial. Procedure-specific exclusions included a history of major abdominal or retroperitoneal surgery, extensive organ resections that altered normal anatomy significantly, and suspicious hilar lymph nodes. Enrollment took place between May 2023 and July 2024. Seven laparoscopic surgeons with various levels of laparoscopic and robotic experience participated. Six of the surgeons were robotic naïve at the start of the study, and the seventh had >5 yr of robotic experience. All surgeons and their OR teams underwent mandatory manufacturer training with DEXTER and completed at least three roll-in cases prior to study participation.

The primary safety endpoint of the study was occurrence of Clavien-Dindo grade III–V complications, up to 30 d postoperatively. The clinical performance endpoint was successful completion of the DEXTER-assisted partial nephrectomy without any device-related permanent conversions. The secondary safety outcomes included the incidence of perioperative (ClassIntra) and early postoperative (Clavien-Dindo) complications, estimated blood loss, rehospitalization, and mortality up to 30 d of follow-up. The secondary performance measures evaluated skin-to-skin operative time, console time, and docking time. Other outcomes of interest included the length of stay, reoperation within 30 d, warm ischemia time (WIT), and clamping techniques.

### 2.1 The DEXTER Robotic Surgery System

The DEXTER Robotic Surgery System consists of four modules: two patient carts with robotic instrument arms, an endoscope cart with a robotic endoscope arm, and an open, sterile surgeon console. The robotic system, including the surgeon console, is covered with sterile drapes and remains sterile throughout the procedure. The endoscope arm is compatible with any endoscope. Five single-use instruments are available: a monopolar hook, monopolar scissors, a bipolar Maryland dissector, a bipolar Johann grasper, and a needle driver. All DEXTER instruments are fully articulated with seven degrees of freedom and can be used with 8-mm trocars.

**Fig. 1:**
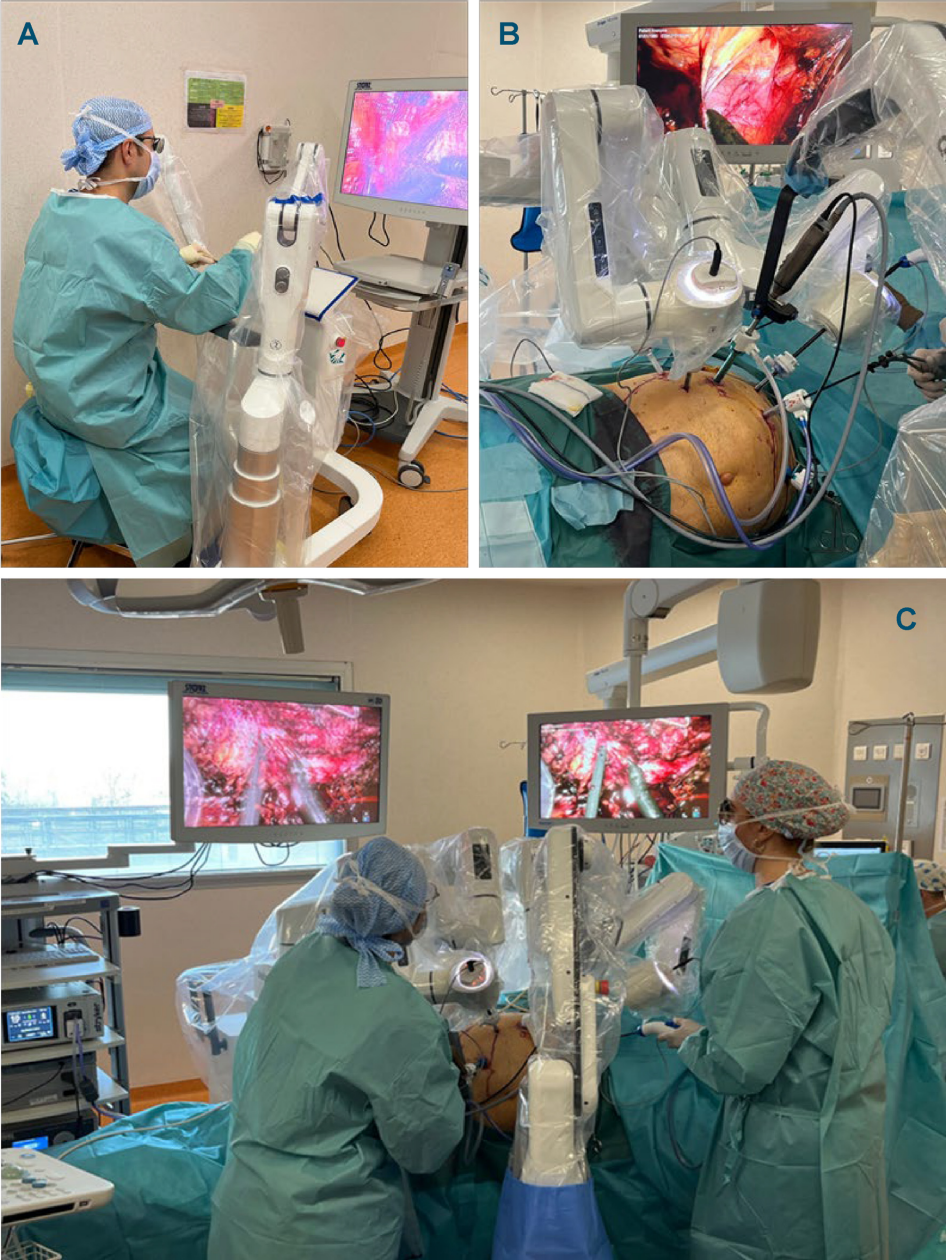
(A) The sterile surgeon at the draped sterile robotic console. (B) Close-up view of patient positioning in lateral decubitus. (C) The OR setup with the three robotic arms docked, and an assistant surgeon and sterile nurse at the patient side. The two patient carts with robotic instrument arms were positioned on the tumor side of the patient, while the robotic camera arm was on the opposite side, next to the assistant. OR = operating room.

DEXTER enables rapid transition between laparoscopic and robotic procedures without redocking, as the robotic arms fold back to provide sufficient space around the patient bed for laparoscopic surgery. As the surgeon is sterile at the console (Fig. 1A), the transition between laparoscopic and robotic modes is instantaneous.

DEXTER is designed to incorporate the full functionality of a laparoscopic tower and is compatible with equipment already present in the OR. In our study, a three-dimensional (3D) Rubina endoscopic system (Karl Storz GmbH, Germany) was used at one site, and 3D Rigid ENDOEYE 30° (Olympus Inc., Japan) was used at the other site*.*

### 2.2 Surgical technique

A four-phase computed tomographic scan was conducted to visualize the tumor location and vascular anatomy to define the tumor approach.

Patients were positioned in a lateral decubitus position (75–80°), with 0° Trendelenburg and a tilt ranging from 0° to 7°. Three translucent laparoscopic trocars were used for the robotic arms (Fig. 1B). The 3D endoscope port was placed in the intersection of the line from the anterior superior iliac spine to the xiphoid process and the perpendicular line that crosses the umbilicus. Two robotic instrument 8/10-mm ports were placed in line with the endoscope port, ensuring a distance of 10 cm between the ports. One 12-mm port was positioned near the umbilicus for the assistant and the use of the endoscopic ultrasound probe. An additional 5-mm trocar was put under the xyphoid when the tumor was on the right kidney, or at 5–10 cm to the right and above the umbilicus when the tumor was on the left kidney (Fig. 1B).

All procedures were conducted with the support of an assistant surgeon and a sterile nurse (Fig. 1C).

The renal hilum was dissected to identify the renal artery and vein. Intraoperative ultrasonography was used to evaluate the tumor border and depth. Three clamping strategies were employed based on tumor characteristics and surgeon preference: no clamping, selective clamping, or full clamping. The choice of performing a clampless procedure was at the surgeon’s discretion based on tumor access and calcification of the renal artery. Resection was performed using robotic monopolar scissors, and the surgical margins were evaluated intraoperatively. The defect was closed using V-Loc 3-0 barbed sutures. The specimen was placed in an endoscopic retrieval bag and extracted through an extended trocar incision. Drain placement was not systematic and left to the surgeon’s discretion.

### 2.3 Data evaluation

All adverse events were reviewed and adjudicated by an independent Clinical Event Committee, who determined whether the events were procedure or device related. Descriptive statistics were used to present the results: median values with interquartile range (IQR). Data analyses were performed using Stata (2023, Stata Statistical Software: Release 18; StataCorp LLC, College Station, TX, USA).

## 3 Results

Between May 2023 and July 2024, 33 consecutive patients (20 male and 13 female patients) were enrolled in the study, with a median age of 70 yr (IQR 60–74), and BMI of 26.2 kg/m^2^ (IQR 23.7–31.2). The American society of Anesthesiologists classification included two patients (6.1%) in class I, 23 patients (70%) in class II, and eight patients (24%) in class III. The median PADUA score was 8 (IQR 6.5–9). Detailed patient characteristics, including indications for surgery, are presented in Table 1.

**Table 1:** Patient characteristics

Parameter	Value
Age (yr), median (IQR)	70 (60–74)
Gender (male), *n* (%)	20 (60)
BMI (kg/m^2^), median (IQR)	26.2 (23.7–31.2)
ASA score, *n* (%)	
I	2 (6.1)
II	23 (70)
III	8 (24)
PADUA score, median (IQR) a	8 (6.5–9.0)
Tumor side (left), *n* (%)	21 (64)
Tumor size (cm), median (IQR) b	3.4 (2.2–4.0)
Preoperative eGFR (ml/min/1.73 m^2^), median (IQR) b	71 (61–89)
Comorbidities, *n* (%)	
Hypertension	16 (48)
Diabetes	6 (18)
Sleep apnea	5 (15)

ASA = American Society of Anesthesiologists; BMI = body mass index; eGFR = estimated glomerular filtration rate; IQR = interquartile range.

a Data collected on 28 patients.

b Data collected on 29 patients.

**Fig. 2:**
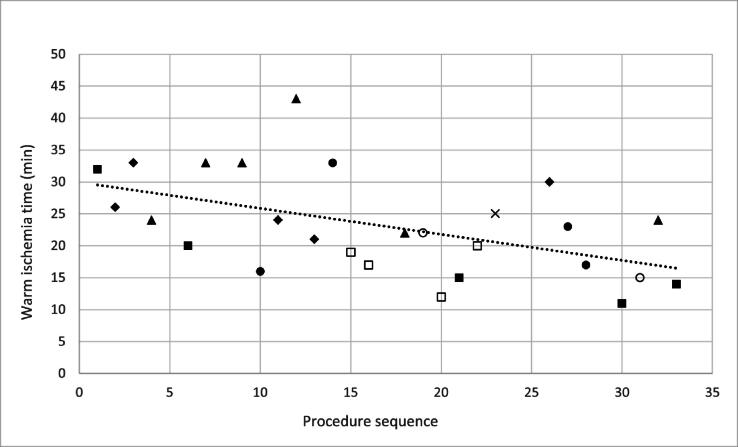
Variation of warm ischemia time in minutes over the series of procedures. Each symbol indicates a different surgeon (▪ = surgeon 1, ◊ = surgeon 2, Δ = surgeon 3. ◦ = surgeon 4, • = surgeon 5, □ = surgeon 6, and × = surgeon 7). The trend line indicates the reduction in WIT over time and experience. WIT = warm ischemia time.

All procedures were completed successfully without permanent conversion to open or laparoscopic surgery. A total of 19 left and 14 right renal masses were removed. The transperitoneal approach was used in 31 patients and the retroperitoneal approach in two patients. Clamping techniques included six clampless, 22 full clamping, and five selective clamping procedures. Among the 27 clamped cases, the median WIT was 20 min (IQR 14–25), showing a declining trend throughout the series (Fig. 2). The median skin-to-skin operative time was 170 min (IQR 137–200), console time was 60 min (IQR 38–87), and docking time was 6 min (IQR 5-7). One minor intraoperative complication occurred (ClassIntra 2 bleeding). The median estimated blood loss was 300 ml (IQR 100–600), with no blood transfusions. The median length of hospital stay was 2 d (IQR 2–3). Perioperative details are available in Table 2.

**Table 2:** Peri- and postoperative results

Parameter	Value
Surgical approach, *n* (%)	
Transperitoneal	31 (94)
Retroperitoneal	2 (6.1)
Conversion to open, *n* (%)	0
Clamping technique, *n* (%)	
No clamping	6 (18)
Selective clamping	5 (15)
Full clamping	22 (67)
Warm ischemia time (min), median (IQR)	20 (14–25)
Conversion to radical nephrectomy, *n* (%)	0
Conversion to laparoscopy, *n* (%)	0
Total operative time (skin to skin; min), median (IQR)	170 (137–200)
Docking time (min), median (IQR)	6.0 (5.0–7.0)
Console time (min), median (IQR)	60 (38–87)
Technique, *n* (%)	
Fully robotic	10 (30)
Robotic and one laparoscopic step	15 (46)
Robotic and two laparoscopic steps	6 (18)
Robotic and three laparoscopic steps	2 (6.1)
Duration of modality switch (s), median (IQR)	10 (7–30)
Estimated blood loss (ml), median (IQR)	300 (100–600)
Blood transfusions, *n* (%)	0
Length of hospital stay (d), median (IQR)	2.0 (2.0–3.0)
Postoperative eGFR (ml/min/1.73 m^2^), median (IQR) a	81 (64–90)
Negative surgical margins, *n* (%) a	29 (100)
Trifecta achieved, *n* (%) a	21 (72)
Patients with serious adverse events, *n* (%)	
Clavien-Dindo IIIa	1 (3.0)
Clavien-Dindo IIIb	2 (6.1)
Clavien-Dindo IV–V	0
Rehospitalization, *n* (%)	3 (9.1)
Reoperation, *n* (%)	2 (6.1)
Mortality, *n* (%)	0

eGFR = estimated glomerular filtration rate; IQR = interquartile range.

a Data collected on 29 patients.

Ten procedures (30%) were performed entirely robotically without any modality switch. A combined laparoscopic-robotic approach was employed for the other surgeries, including one modality switch in 15 cases (46%), two modality switches in six cases (18%), and three modality switches in two procedures (6.1%). The mean time required to switch to laparoscopy was 10 s per switch (IQR: 7–30), varying according to the step performed by the surgeon at the patient bedside (Table 2). One device malfunction occurred during the study related to a needle holder instrument that was not recognized by the system and therefore replaced. There was no disruption to the surgical flow and no adverse events were associated with the device malfunction.

Regarding 30-d postoperative outcomes (Table 2), no device-related adverse events occurred. Fourteen Clavien-Dindo grade I events were reported in nine patients (27%), and five grade II events were reported in four patients (12%). Grade III complications were noted in three patients: one IIIa and two IIIb. The IIIa complication was a false aneurysm with a urinary tract fistula, requiring selective embolization, without reoperation. The first IIIb complication was a left kidney urinoma, necessitating JJ placement and percutaneous radiological drainage. The second complication involved an infected urinoma without bacteremia, requiring rehospitalization and endoscopic JJ catheter placement. No grade IV or V events occurred. The renal function was preserved with a median ΔeGFR of –1 ml/min/1.73 mm^2^ (IQR: –10.5 to 7.2) between the preoperative and the 30-d postoperative assessments.

All surgical margins were negative, and trifecta was achieved in 72% of the cases.

DEXTER provided dexterity and precision in key surgical steps, particularly during tumor resection and renorrhaphy.

## 4 Discussion

This is the first prospective multicenter study on DEXTER-assisted RAPN, involving seven surgeons across two French hospitals. The aim was to collect early real-world evidence on safety and clinical performance. Most surgeons were at the beginning of their robotic learning curve, with six being robotic naïve and performing their first robotic procedures during the study. Nevertheless, all procedures were completed successfully without conversion to open or radical nephrectomy.

The instinctive design of DEXTER and laparoscopic-like setup provided a familiar environment transitioning to robotics. The three-arm configuration proved adequate for RAPN, consistent with previous comparisons between three- and four-arm configurations on other systems [20], [21], [22], [23]. Its LAP mode, where arms retract compactly, allowed smooth alternation between robotic and laparoscopic steps, with transitions averaging only 10 s. The open console design supported direct communication with the team, particularly valuable for guiding intraoperative ultrasound use. Open console design has been shown to improve team cohesion and workflow, and increase surgeon availability in the OR environment [24]. These factors are of high importance during the learning curve of a robotic system. DEXTER offers a simple setup, compatibility with standard laparoscopic equipment, and minimal infrastructure requirements, allowing efficient workflow in a familiar environment. Owing to the quick transitions between laparoscopic and robotic modalities, DEXTER offers a flexible solution for diverse surgical environments.

In addition to workflow advantages, the cost considerations of DEXTER are favorable. The acquisition price is 15–30% lower than that of conventional robotic platforms, and the single-use instruments remove the need for reprocessing. DEXTER’s modular, mobile design, along with its compatibility with the existing OR equipment, further reduces logistical and financial barriers to adoption.

WIT is a critical parameter in partial nephrectomy, with 25 min generally considered safe [25]. In this study, median WIT was 20 min, decreasing over time as surgeons gained experience. This is within safe limits and in a similar range to the published values for da Vinci (12–30.4 min) [26], [27], [28] and HUGO-RAS (9.9–18.9 min) [29], [30], [31], despite the absence of prior robotic expertise in our cohort.

The inclusion of tumors with varied PADUA scores confirmed feasibility across different complexities. Operating times were reasonable, and no device-related complications occurred. Overall complications were frequent but mostly minor, while major complications (9%) were procedure related and comparable with reported ranges for other robotic platforms (3.6–19%) [32], [33], [34].

The main principles of an Enhanced Recovery After Surgery (ERAS) protocol were implemented as per the recommendations of the French Urology Association [35], such as not administering morphine derivatives, and not using a drain or early drain removal, allowing a short median length of stay of 2 d in the cohort. The low conversion rates, few serious complications, and preservation of the early postoperative renal function support the safety and usability of DEXTER in RAPN.

Despite its prospective multicenter design, the study had limitations: learning curve assessment was hampered by a low case load per surgeon, and the small sample size limits generalizability. Nonrandomized patient selection and absence of a control group, with comparisons made with only published data on similar devices, reduce the possibility of a comparative performance evaluation. Short-term oncological outcomes were not assessed, and follow-up was limited to 30 d, restricting the evaluation of long-term functional and oncological outcomes. In addition, none of the surgeons performed more than seven cases, which is insufficient to represent the initial learning phase for RAPN [36], [37], making it impossible to evaluate the learning curve for DEXTER.

A major limitation of this study is the absence of predefined criteria for arterial clamping, resulting in a potential selection bias. The choice between clamped and clampless techniques was based on intraoperative surgeon judgment rather than objective preoperative parameters. As a result, patients selected for clampless procedures may have had more favorable tumor characteristics, which could influence the perioperative and functional outcomes. This limitation is inherent to retrospective and exploratory studies reflecting real-world surgical practice. Therefore, comparisons between clamped and clampless approaches should be interpreted cautiously, and the results should not be considered as evidence of superiority of one technique over the other.

Larger studies are needed to validate these findings and further explore the benefits of DEXTER in urology. As clinical adoption expands, real-world data will become available to support its safety and performance, including outcomes beyond the initial cases. Future studies will also be required to document the learning curve of DEXTER based on prior experience of the user and a cost analysis of the use of DEXTER in RAPN.

## 5 Conclusions

This multicenter study supports the short-term safety and procedural feasibility of DEXTER as an effective platform for RAPN. Clinical performance and major complication rates were consistent with the established practice standards. Importantly, the procedures were performed during the surgeon’s initial cases, highlighting the instinctive design of DEXTER. Its main advantage lies in its seamless integration into the existing laparoscopic OR workflow, facilitating transition to robotic surgery. Larger-cohort studies with longer follow-up are needed to confirm these results and evaluate long-term patient outcomes in routine clinical practice.

  ***Author contributions*:** Guillaume Hugues had full access to all the data in the study and takes responsibility for the integrity of the data and the accuracy of the data analysis.

  *Study concept and design*: Hugues.

*Acquisition of data*: Thillou, Robin, Forgues, Benali, Emeriau, Durand, Hugues.

*Analysis and interpretation of data*: Hugues.

*Drafting of the manuscript*: Hugues.

*Critical revision of the manuscript for important intellectual content*: Thillou, Robin, Forgues, Benali, Emeriau, Durand, Hugues.

*Statistical analysis*: None.

*Obtaining funding*: None.

*Administrative, technical, or material support*: None.

*Supervision*: Hugues.

*Other*: None.

  ***Financial disclosures:*** Guillaume Hugues certifies that all conflicts of interest, including specific financial interests and relationships and affiliations relevant to the subject matter or materials discussed in the manuscript (eg, employment/affiliation, grants or funding, consultancies, honoraria, stock ownership or options, expert testimony, royalties, or patents filed, received, or pending), are the following: All authors: Distalmotion—editorial and journal submission advice. Nadia Ali Benali: Worldone Research Ltd. (expert testimony); Laboratoires Bouchara Recordati, Sanofi Winthrop Industrie, Laboratoires Coloplast, and Astellas Pharma (support for attending meetings). Xavier Durand: MSD France (support for attending meetings). Damien Emeriau: Distalmotion (expert testimony); Pierre Fabre Medicament, Laboratoires Bouchara Recordati, Ipsen Pharma, Astellas Pharma, Janssen-Cilag, Laboratoires Besins International, Coloplast, Distalmotion, Sanofi Winthrop Industrie, Hollister France Inc., FSK, Accord Healthcare France, and Viatris France (support for attending meetings). Aurélien Forgues: Distalmotion (expert testimony); Laboratoires Coloplast, Ipsen Pharma, Astellas Pharma, and Janssen-Cilag (honoraria); MSD France, Janssen Cilag, Coloplast, Accord Healthcare, Astellas Pharma, Distalmotion, Hollister France Inc., Ipsen Pharma, Laboratoires Besins International, Laboratoires Bouchara Recordati, Lilial, Pierre Fabre Medicament (support for attending meetings). Guillaume Hugues: Distalmotion (expert testimony, consulting, honoraria, and support for attending meetings); M3 Global research Ltd., B3TSI, and Athenum partners GmBH (expert testimony); Distalmotion, Accord Healthcare, Astellas Pharma, Exafield, FSK, Hollister France Inc., Ipsen Pharma, Laboratoires Besins International, Laboratoires Bouchara Recordati, Laboratoires Coloplast, Lilial, Pascaleo, Pfizer SAS, Pierre Fabre Medicament, Sanofi Aventis France, Sanofi Winthrop Industrie, Teleflex Medical, and Viatris Santé (support for attending meetings). Coline Ricolleau: FSK and Ipsen Pharma (support for attending meetings). Humphrey Robin: Distalmotion (consulting fees); Accord Healthcare, Astellas Pharma, Bristol Myers Squibb, Distalmotion, FSK, Hollister France Inc., Ipsen Pharma, Janssen-Cilag, Laboratoires Besins International, Laboratoirs Bouchara Recordati, Laboratoires Coloplast, Pierre Fabre Medicament, Sanofi Aventis France, and Sanofi Winthrop Industrie (support for attending meetings). Damien Thillou: Distalmotion (consulting fees, expert testimony, honoraria, and support for attending meetings); Accord Healthcare France, Astellas Pharma, Besins Healthcare France, Diadom, Hollister France Inc., Ipsen Pharma, Janssen-Cilag, Laboratoires Besins International, Laboratoires Bouchara Recordati, Laboratoires Coloplast, Lilial, Pierre Fabre Medicament, Sanofi, and Teleflex Medical (support for attending meetings).

  ***Funding/Support and role of the sponsor*:** Distalmotion is the sponsor of this study and was responsible for study concept and design, management of data, data analysis, and review of the manuscript. The sponsor played a role in the design and conduct of the study, management and analysis of the data, and review of the manuscript.

  ***Acknowledgments*:** The authors wish to acknowledge the contributions of Sylvia Daunas, study nurse, and Dr. Coline Ricolleau, surgical assistant.

  ***Data sharing statement*:** Deidentified participant data from this study may be shared upon reasonable request, with appropriate data sharing agreement in place.
